# Influence of Fe_x_O_y_ and Al_2_O_3_ Contents on the Thermal Stability of Iron Ore-Waste Fibers: Key Mechanisms and Control

**DOI:** 10.3390/ma17143480

**Published:** 2024-07-14

**Authors:** Xiaoguang Li, Xiaohui Wang, Xianju Fang, Xianglong Shen, Liding Huang, Jinyi Qin, Wanzhang Fu, Weiguang Li

**Affiliations:** 1 College of Civil Engineering, Chang’an University, Xi’an 710061, China; wxhui@163.com (X.W.); 15686200163@163.com (X.F.); sxl2190@163.com (X.S.); 19137772982@163.com (L.H.); jinyi.qin@chd.edu.cn (J.Q.); fwz@chd.edu.cn (W.F.); liwg@mail.nwpu.edu.cn (W.L.); 2 College of Aviation, Northwestern Polytechnical University, Xi’an 710072, China

**Keywords:** iron-ore waste, Fe_x_O_y_, bridged oxygen, glass transition temperature, thermal stability

## Abstract

Traditional rock wool fibres are susceptible to crystallization and pulverization. To mitigate this, glass fibres were produced from iron ore waste (IOW). When the ratio of Fe^2+^ and Fe^3+^ is 1:3 and the Al_2_O_3_ content is 10 wt.%, increasing the Fe_x_O_y_ content enhances the thermal stability.At an Fe_x_O_y_ content of 17–19% and an Al_2_O_3_ content of 10–13%, the glass transition temperature (Tg) peaked. Increasing the Fe_x_O_y_ content from 10% to 20% enhanced the stability of Si-O and Al-O bonds and increased bridged oxygen, stabilizing the structure. Here, Fe^2+^ balances structural charges, while Fe^3+^ replaces some Al atoms in the network. When the Al_2_O_3_ content is 10–13% and the Fe_x_O_y_ content is 17–19%, the thermal stability of the IOW rock glass reaches its optimal level. At 20% Fe_x_O_y_ content, the structure becomes three-dimensional and cyclic, increasing polymerization. Consequently, incorporating Fe_x_O_y_ alongside a 10% Al_2_O_3_ content improves thermal stability, supporting the development of high-stability rock wool from IOW. This approach also enhances the refractory properties of rock wool fibres within the Fe_x_O_y_-Al_2_O_3_-SiO_2_-MgO-CaO system.

## 1. Introduction

Traditional rock wool fibres are made from basalt and consume natural resources. When heated by fire, they are prone to crystallisation and pulverisation, resulting in decreased strength. Therefore, long-term use of such fibres poses a safety hazard [1,2]. However, the acidity coefficient of iron ore waste (IOW) is close to that of basalt for the production of rock wool; thus, it can potentially be used as a raw material for the production of rock wool fibres [3,4]. IOW is rich in Fe and Al, which improves the thermal stability of glass [5]. Therefore, the use of IOW to produce rock wool may replace traditional rock wool to avoid crystallisation and powdering during fires [6].

Al_2_O_3_ is a typical amphoteric oxide that exhibits acidity in alkaline melts [7]. Forming a [AlO_4_] tetrahedral structure through covalent bonding between Al and O leads to the formation of a three-dimensional network structure that acts as a network-forming agent. In the acid melt, Al_2_O_3_ becomes alkaline, and an ionic bond is formed between Al and the anion, acting as a network modifier [8]. In the (CaO-SiO_2_)_(1-x)_(Al_2_O_3_)_x_ system where x = 0–30 mol%, an increase in the population of bridging oxygens and tricluster oxygens are observed with increasing Al_2_O_3_ content, while the number of free oxygens tends to diminish. Regions with a high number of Al-O-Al linkages start to appear, which is also accompanied by an increase in the glass transition temperature and elastic moduli [9]. However, in the CaO-Al_2_O_3_-SiO_2_ system, a concentration of Al_2_O_3_ more than 30% leads to a relative deficiency of Ca atoms and an increase in tri-coordinate O and five-coordinate Al content. This can lead to an increase in the self-diffusion coefficients of Al and O atoms, and the structure tends to become unstable [10]. IOW rocks comprise a complex Fe_x_O_y_-Al_2_O_3_-SiO_2_-MgO-CaO(FASMC) system, with Fe^3+^ and Fe^2+^ coexisting in aluminosilicate glasses playing different roles in the microstructure [11].When the mass fraction of Al_2_O_3_ is 15 wt.%., increasing the Fe_2_O_3_ content from 2 wt.%. to 22 wt.%. provides charge compensation for the [AlO_4_] tetrahedron, leading to a decrease in the coordination number of the Al-O bond and the formation of a more stable 4-coordinate Al structure [12]. With the increase in FeO content from 5 wt.%. to 25 wt.%., free oxygen O^2−^ ions destroy the Si-O bond, leading to the depolymerization of the aluminosilicate structure. When the FeO content exceeds 23 wt.%.,the Al-centred tetrahedral structure in the SiO_2_-Al_2_O_3_-FeO system is depolymerized easily [13]. The relative ratio of Fe^3+^ to Fe^2+^ determines the degree of polymerisation within the glass structure [14,15].

Controlling the changes in the Fe_x_O_y_ and Al_2_O_3_ contents provides a feasible approach to preparing highly thermally stable IOW fibres, which is a new way to improve the refractoriness of rock wool fibres based on the FASMC system. Existing studies have focused primarily on the effect on aluminosilicates when the Fe_x_O_y_ content is low or only in a single valence state. There is a gap in the theoretical study of IOW fibres based on the FASMC structure regarding the influence of Fe_x_O_y_ and alumina content in the range of 10–20% on the overall structure.

In this study, X-ray photoelectron spectroscopy (XPS) was used to analyse the detailed composition of IOW rocks for formula design, and a structural unit model was established. The roles of Fe_x_O_y_ and Al_2_O_3_ in the microstructure were analysed using molecular dynamics (MD) simulations. By comparing the measured and simulated data, we investigated the correlation between the changes in Fe and Al contents with the glass transition temperature (T_g_) and thermal stability and verified the reliability of the simulation results. In order to optimise the thermal stability of the FASMC structures, the effects of iron and aluminium content on the radial distribution function (RDF), coordination number (CN), and bridging oxygen (BO) of the glass structures were analysed. Finally, Raman spectroscopy was used to confirm the effect of the Al/Fe content in the structure on the degree of polymerisation. These promising results provide a theoretical basis for the production of fibres with high thermal stability by controlling the Al/Fe content of IOW rocks.

## 2. Materials and Methods

### 2.1. Component of IOW Rocks

**Figure 1:**
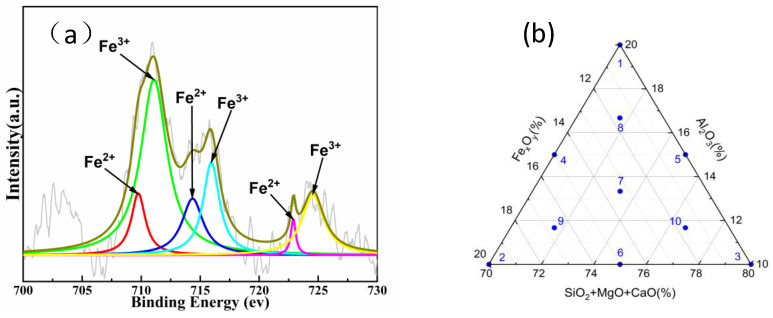
(**a**) XPS graph of IOW rocks and (**b**) formula design based on the simplex method.

IOW rocks with Fe_x_O_y_ and Al_2_O_3_ contents in the range of 10–20% were tested using XPS (250 xi, Thermo Fisher, Waltham, MA, USA). The fitted results are shown in Figure 1. 

As shown in Table 1, Fe^2+^/Fe^3+^ = 1/3. Therefore, when constructing a microstructural model of the IOW rocks, the ratio of Fe^2+^ to Fe^3+^ in Fe_x_O_y_ should be 1/3.

### 2.2. Molecular Dynamics Simulation of Glass Based on IOW Rocks

#### 2.2.1. Preset Parameters for Simulation Analysis

The initial model was constructed using Materials Studio, and the proportions of raw material components were obtained using the simplex method, as shown in Table 2.

For the simulation, the Born–Mayer–Huggins potential function, which comprises the van der Waals force, long-range Coulomb interaction, and short-range repulsive interaction, was chosen to describe the interactions of each atomic pair [16]. The Fe-O parameters were obtained from Belashchenko [17,18], and the other parameters were obtained from the literature [19,20,21]. In the simulation, each model had 6000 atoms with a density of 2.7 g/cm^3^. The atoms were randomly placed in a model box. Creating an infinite system without boundaries using three-dimensional periodic boundary conditions yielded more accurate results. The truncation radius was set to 12.5 Å for the short-range force, the long-range Coulomb force was calculated using the Ewald summation method, the MD was calculated using the Verler velocity algorithm with an integration step of 1 fs, and the temperature and pressure were based on the Nose–Hoove and Barostat methods [21].

#### 2.2.2. Molecular-Dynamics Simulation Process

The system was relaxed to 6000 K for 100 ps to remove the effects of the initial state of the atoms. The system was then cooled to 1773 K in 80,000 steps and finally relaxed for 500 ps at 1773 K before being further quenched from 1773 K to 300 K over 1 ns and equilibrated at 300 K for 500 ps to ensure that the atoms in the system were well mixed so that structural information, such as the RDF [22,23] and CN [24], could be obtained. The Neumann–Planck–Thompson ensemble was selected, and the structure was gradually heated from 300 K to 1500 K over 500 ps to obtain information such as the glass transition temperature. The density curves of the last smooth section at each temperature were averaged.

### 2.3. Glass Structure Preparation

**Figure 2:**
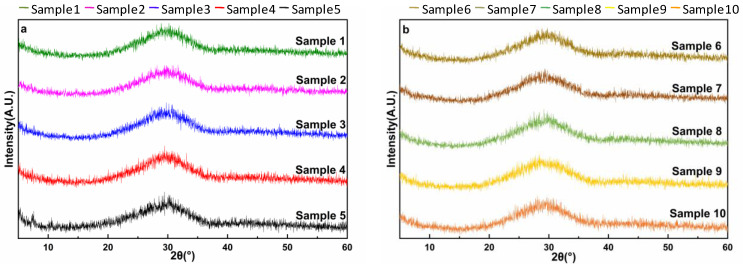
XRD patterns of prepared IOW glass. The figure (**a**,**b**) shows the XRD diffraction patterns of 10 groups of vitreous, the diffraction patterns of the samples show a wide hump in the range of 20–35°, and there is no obvious mineral crystalline phase appearing in the ten samples, which has good vitreous characteristics, indicating that the vitreous samples were successfully prepared.

Based on the proportions of the oxides listed in Table 2, the materials were fully ground with a sample grinder for 10 min and placed in a 100 mL alumina crucible and melted uniformly at 1500 °C for 1 h. The furnace chamber was opened and the sample was taken out together with the crucible and placed in cold water to obtain the samples. The vitreous sample melts at high temperatures and cools rapidly, resulting in a glassy state. The vitreous sample was dried and ground to a powder in a sample mill. The glass was analysed by X-ray diffraction (XRD), as shown in Figure 2. No significant sharp peaks were observed in the crystalline phase, nor in the crystalline phase. Within the characteristic diffraction peak range of 20–35°, a clear amorphous characteristic envelope was observed, indicating that the sample has fine glass transition characteristics.

### 2.4. The Characteristics of Glass

The produced glass was thoroughly ground to a powder and then passed through a 200 mesh sieve for structural testing. It was added to a crucible and heated from room temperature to 1200 °C at a rate of 10 °C/min under nitrogen atmosphere. The Tg values were simulated with a simultaneous thermal analyser (TG-DSC, SDT Q600, TA, USA) and measured by isobaric method [25]. Five groups of glass were selected with ratios of 10% Al_2_O_3_ and 10%, 11.67%, 15%, and 20% Fe_x_O_y_ for Raman spectroscopy analysis (Raman, Alpha300R, WITec, Ulm, Germany) [26]. The results were obtained using Gaussian decomposition within the range of 800–1200 cm^−1^. The integral area of the peaks was used to evaluate the content of the characteristic structural units in the system.

## 3. Results and Discussions

### 3.1. The Glass Transition Temperature (T_g_)

T_g_ is an important parameter reflecting the thermal stability of a material [27]. Currently, no unified explanation for this glass transition phenomenon exists [28]. The free volume theory states that a molecular motion energy below T_g_ is low, resulting in frozen chain segments. With increasing temperature, the change in the free or void volumes of the polymers was very small. During the heating process, the particles within the rock wool fibre system were rearranged, causing the ion bonds to break and reform, thereby leading to crystallisation. Consequently, the volume of fibres expanded, causing crystallisation and powdering. 

**Figure 3:**
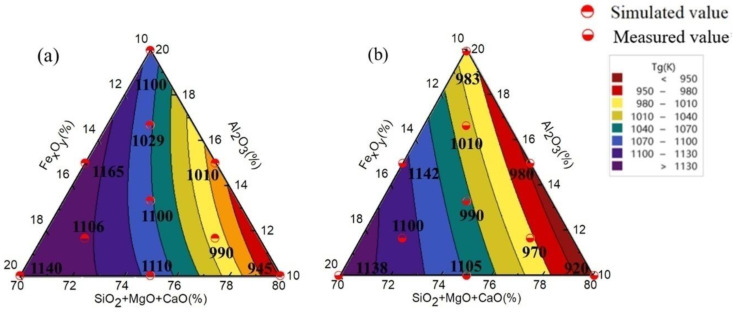
(**a**) Simulated T_g_ and (**b**) measured T_g_ thermal analysis of IOW fibres based on FASMC structure.

MD simulations can predict the initial crystallisation and pulverisation temperatures of rock wool fibres, thereby determining the T_g_ value of the material and characterising the thermal stability of the microstructure. The higher T_g_ value, the better the thermal stability and fire resistance of the rock wool fibres. The T_g_ values simulated and measured by MD are shown in Figure 3a,b.

As shown in Figure 3a, when the Al_2_O_3_ content is relatively low and the Fe_x_O_y_ content increases from 10% to 20%, T_g_ increases by 20% to 30%, resulting in a corresponding increase in thermal stability. As the Fe_x_O_y_ content increases, Fe^3+^ forms a tetrahedral structure of [FeO_4_] to compensate the discontinuous network in the glass matrix. Simultaneously, Fe^2+^ acts as an alkaline earth metal ion and provides charge compensation. This forms more tetrahedral structures, thereby making the structure more stable. The highest range of the T_g_ was 1125–1165 K, with the Fe_x_O_y_ and Al_2_O_3_ contents ranging from 15% to 20% and 10% to 15%, respectively.

The measured T_g_ values are shown in Figure 3b. When the Al_2_O_3_ content is maintained, the T_g_ value increases with increasing Fe_x_O_y_ content. When the Fe_x_O_y_ and Al_2_O_3_ contents are between 17 and 20% and 10 and 13%, respectively, the T_g_ ranges from 1130 K to 1142 K. A comparison of Figure 3a and b shows that the experimental value is marginally lower than the simulation value. This may be because the MD simulation time is relatively short, whereas the heating rate of the experimental environment gradually increases in seconds, and the movement of elements is more abundant. This implies that the molecular chain segments in the polymer material move more smoothly at high temperatures, and the intermolecular forces weaken. The movement of polymer segments makes exceeding the critical temperature easier, resulting in a decrease in the T_g_ [29]. Therefore, the measured T_g_ value was marginally lower than the simulated value. In summary, the variation trends in the T_g_ obtained from the simulation and measurement were consistent.

**Figure 4:**
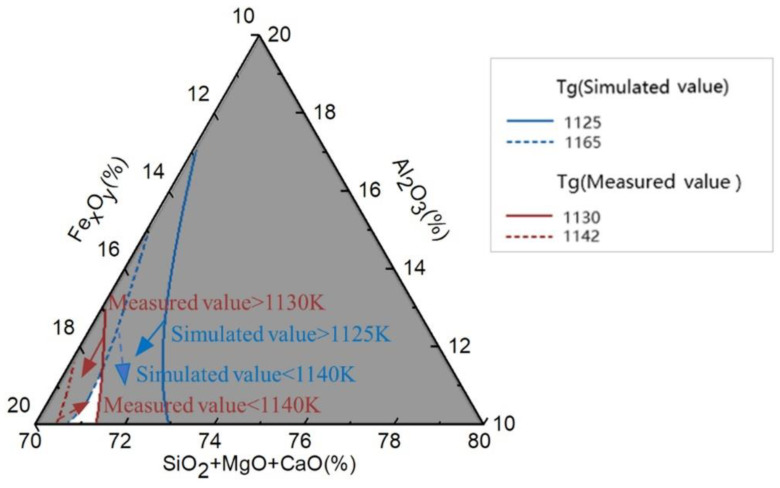
Superimposed contour plot of simulated and measured values.

To obtain the highest T_g_, the ranges of the simulation and measurement results were overlaid, as shown in Figure 4. The highest T_g_ is indicated by the white area, with Fe_x_O_y_ and Al_2_O_3_ contents ranging from 17% to 19% and 10% to 13%, respectively. Within this T_g_ range, the IOW rock wool exhibited the best thermal stability.

### 3.2. Bond Length Simulation of Atomic Pairs

**Figure 5:**
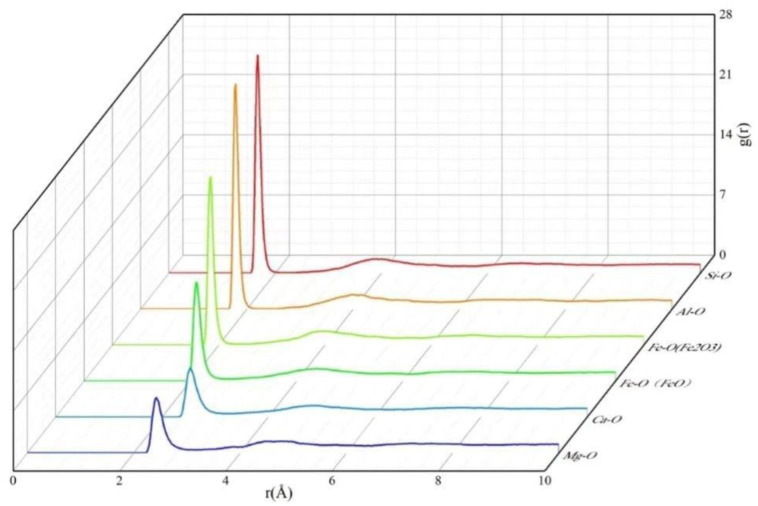
The average RDFs of atom bonds.

Figure 5 shows the average bond length of each atom in an RDF diagram. The average bond length is the horizontal coordinate of the first peak in the RDF of the corresponding atom, representing the nearest neighbour bond length of the atom pair. A narrow and sharp RDF peak indicates that the atomic bond formed between the two atoms is more stable. The nearest-neighbour bond lengths of various atomic pairs are listed in Table 3.

Figure 5 shows that the bond length of Fe-O(Fe_2_O_3_) was close to that of Al-O, suggesting that Fe-O(Fe_2_O_3_) can repair a network structure similar to that of Al-O and that Fe (Fe_2_O_3_) and Al can be transformed into each other. The Ca-O, Mg-O, and Fe-O(FeO) peaks were wide and weak, mainly because Ca^2+^, Mg^2+^, and Fe^2+^ acted as modified ions in the silicate network structure. The Al-O bond lengths ranged from 1.745 to 1.785, which may have been due to the coordination of Al with Al^IV^, Al^V^, and Al^VI^ in the structure [30,31,32]. These coordination states affected the length of the Al-O bond. Table 3 shows that the Ca-O bond length is shorter than that of Mg-O. This may be due to the fact that Ca^2+^ has a larger radius than Mg^2+^ which has a wider range of structural activity. According to the RDF plot, the average bond lengths of Si-O, Al-O, Fe-O(Fe_2_O_3_), Fe-O(FeO), Ca-O, and Mg-O are close to the experimental values [27], indicating the reliability of the simulated results. More importantly, in the simulation of the glass, the first peak of Si-O is narrow and sharp, indicating that the short-range structural unit of Si-O is stable, whereas the structural unit stability of Al-O ranks second.

**Figure 6:**
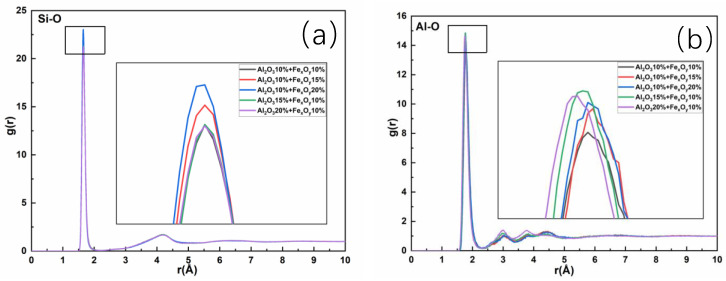
RDF graphs of (**a**) Si-O and (**b**) Al-O.

Five sets of samples with Al_2_O_3_ contents within 10–12% and Fe_x_O_y_ contents within 10–20% were selected to simulate the RDFs of Si-O and Al-O, as shown in Figure 6. When the Al_2_O_3_ content is between 10% and 12%, and Fe_x_O_y_ content increases from 10% to 20%, the RDF peak of Si-O exhibits a significant upward trend. When the Fe_x_O_y_ content reaches 20%, the RDF peak reaches its maximum value. This indicates that within the range of Al_2_O_3_ content, the stability of Si-O bonding can be improved by increasing the Fe_x_O_y_ content. A closer look at the changes in the Al-O bonds showed that as Fe_x_O_y_ content increases, the average bond length and RDF peak increase. Similarly, the high RDF peak corresponding to 20% Fe_x_O_y_ indicates the enhanced stability of Al-O. Because Fe_x_O_y_ in glass mainly exists in the form of Fe^3+^, it substitutes some Al atoms and becomes part of the network structure. Therefore, a moderate increase in Fe_x_O_y_ can enhance the stability of Si-O and Al-O bonds and promote an increase in the T_g_.

### 3.3. Coordination Number Simulation of Atomic Bonds

The CNs of each atom were obtained by integrating the RDF curve. The wider and flatter the CN curve, the more stable the atomic bond. 

**Figure 7:**
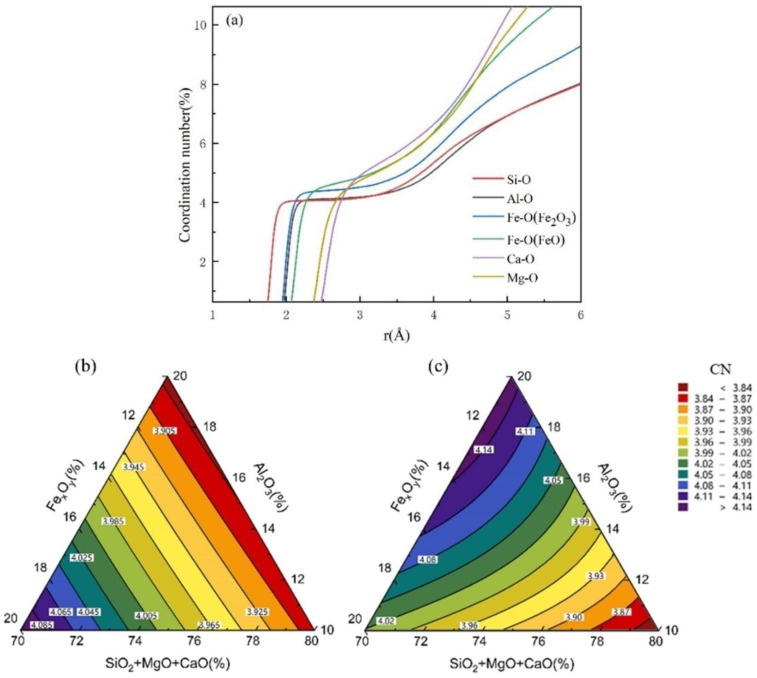
(**a**) The average CN curves in the system; (**b**) Si-O curves and (**c**) Al-O curves of CNs.

Figure 7a showed that the average CNs of Si-O, Al-O, Fe-O(Fe_2_O_3_) and Fe-O(FeO), Ca-O, Mg-O were 3.99, 4.13, 4.50, 5.14, 5.46, and 5.23, respectively. These results are consistent with those previously reported [33,34,35]. As is well known, the most stable structure in the aluminosilicate network is the tetrahedron. That is, the closer the CNs of Si-O or Al-O are to 4, the more stable the structure. Figure 7b shows that regardless of the changes in the Al_2_O_3_ and Fe_x_O_y_ contents, the CN of Si-O remains at approximately 4. The corresponding experimental value is 3.99 Å, with a relative error of only 0.02% [36]. This indicates that the four-coordination structure of Si-O is stable. As shown in Figure 7c, when the Al_2_O_3_ content is between 10% and 12%, Fe_x_O_y_ content increases from 10% to 16%, and the CN of Al-O increases from 3.84 to 4.01. When the Fe_x_O_y_ content increases from 16% to 20%, the CN of Al-O exceeds 4. Therefore, when the content of Fe_x_O_y_ is 16%, the Al-O structure is the most stable.

In conclusion, the stability of Si-O is higher than that of Al-O because the bond length of Fe-O (Fe_2_O_3_) was close to that of Al-O, suggesting that Fe-O (Fe_2_O_3_) can repair a network structure similar to that of Al-O and it replaces some Al atoms to some extent. As the Fe_2_O_3_ content increases, the stability of the Si-O and Al-O bonds improves. Simultaneously, the CN of Si-O remains at approximately 4, indicating a dense structure with a high thermal stability.

### 3.4. Simulation of the Distribution of Bridge Oxygen (O_b_) in the Atomic Bonds

**Figure 8:**
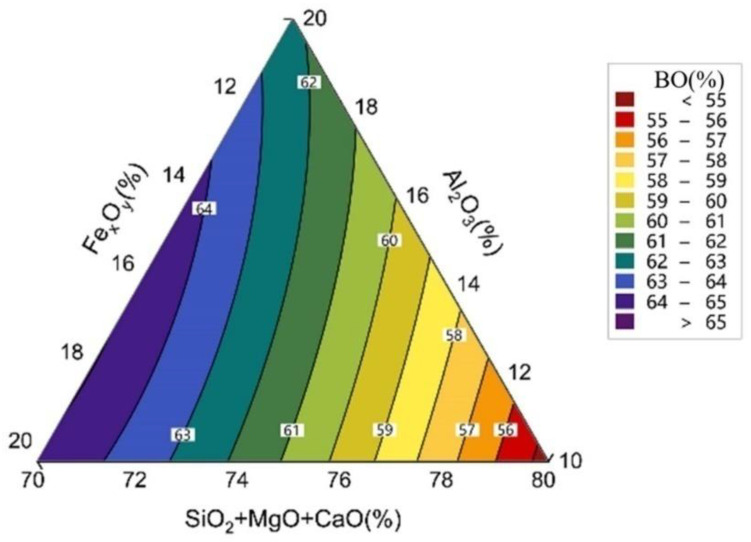
The effect of Fe_x_O_y_ and Al_2_O_3_ changes on bridge oxygen.

The types of oxygen present in conventional silicate structures are categorised as O_b_, non-bridged oxygen (O_nb_), and free oxygen (O_f_), which are in dynamic equilibrium [26,37,38]. O_b_ is assumed to connect the two polymer structures. In other words, both ends of O_b_ are connected to tetrahedral structures. This study includes six types of O_b_: Si-O_b_-Si, Si-O_b_-Al, Al-O_b_-Al, Si-O_b_-Fe(III), Al-O_b_-Fe(III), and Fe(III)-O_b_-Fe(III). The O_b_ content represents the degree of polymerisation within the system; the higher the O_b_ content, the closer the reticular structures are linked to each other. The distribution of O_b_ in the FASMC structure is shown in Figure 8.

As shown in Figure 8, when the content of Al_2_O_3_ remains constant, the O_b_ content gradually increases with increasing Fe_x_O_y_ content. This is because the Fe^2+^ in Fe_x_O_y_ balances the charge in the structure, providing the possibility for the formation of tetrahedra. However, Fe^3+^ in Fe_x_O_y_ acts as a ligand to strengthen the connection between the tetrahedra, forming more [FeO_4_], thereby gradually increasing the O_b_ content and stabilising the structure. When the Al_2_O_3_ content is 10–12% and the Fe_x_O_y_ content is greater than 14%, the structure has a high amount of O_b_ with a higher degree of structural polymerisation. This may be because Al_2_O_3_, as an acidic oxide, forms a network structure with oxygen, and the essence of acidic oxides is the formation of functional groups by acidic cations and oxygen ions, thereby increasing the degree of polymerisation and O_b_ content. This enhances the structural stability of the system. In summary, when the Fe_x_O_y_ content is 14–20% and the Al_2_O_3_ content is 10–12%, the highest content of O_b_ in the microstructure is 64%, resulting in a more stable structure and a higher T_g_.

### 3.5. Characteristic Raman Spectroscopy of Glass

The characteristic peak near 850 cm^−1^ represents the Q^0^(Si) group, which is connected to four non-bridged oxygen atoms on each network-forming atom. The peaks in the region of 900–1190 cm^−1^ can be sequentially represented as the characteristic peaks of the Q^1^(Si), Q^2^(Si), Q^3^(Si), and Q^4^(Si) groups [39,40,41,42]. The band in the region of 1120–1190 cm^−1^ represents Q^4^(Si), which is associated with the complete aggregation of the network structure [1].

**Figure 9:**
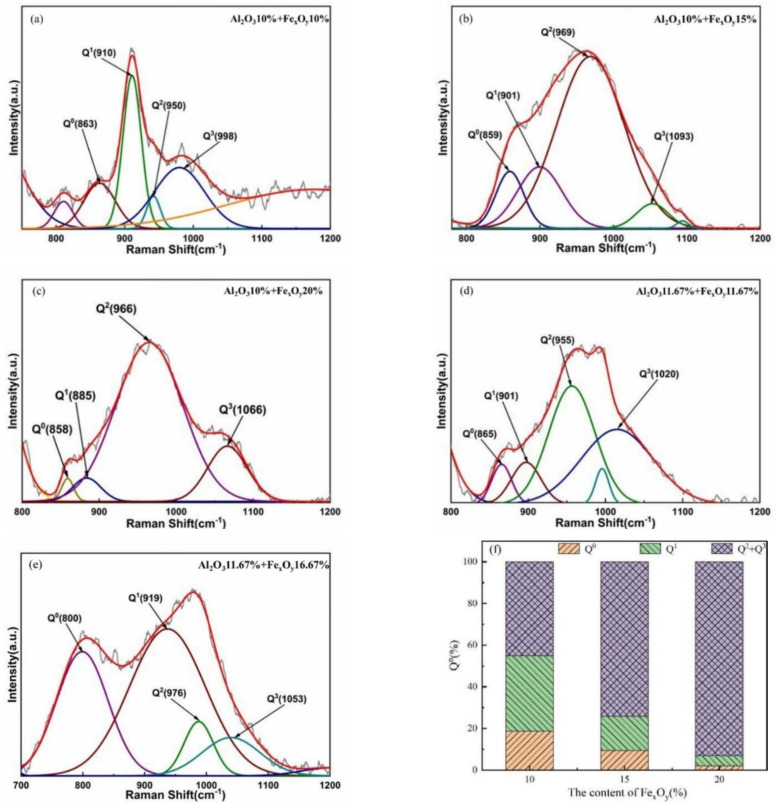
(**a**–**e**) Peak fitting results in the range of 800–1200 cm^−1^ for Raman spectroscopy. (**f**) Peak area proportion of each group at Al_2_O_3_ content of 10–12% and Fe_x_O_y_ content increased from 10% to 20%.

Figure 9 shows the integration of the characteristic peaks. As no obvious characteristic peaks of Q^4^(Si) were obtained in any of the samples, only the percentages of Q^0^(Si), Q^1^(Si), and Q^2^(Si)+Q^3^(Si) are displayed. When the Al_2_O_3_ content is between 10% and 12%, the relative strengths of Q^0^(Si) and Q^1^(Si) gradually decrease with an increase in Fe_x_O_y_ content. Q^1^(Si) represents the connection of three non-bridged oxygen atoms to each network-forming atom. Q^0^(Si) and Q^1^(Si) have simple polymer structures. When the content of Q^0^(Si) and Q^1^(Si) is lowered, they may form looser structures such as chains, layers, or fibres. This is mainly because a lower content results in weaker interaction forces, which loosen the structure. As the Fe_x_O_y_ content increases, the relative strength of Q^2^(Si)+Q^3^(Si) increases, and the vibration near Q^2^(Si) is caused by the vibration of each silicon containing two non-bridged oxygen atoms in the network-structure unit. The band near Q^3^(Si) is the result of the symmetrical vibrations in the network structure unit, where each network-forming atom is connected to only one non-bridged oxygen atom. When the content of Fe_x_O_y_ is 20%, the relative intensity of the Q^2^(Si)+Q^3^(Si) group is the highest. Q^3^(Si) typically represents complex polymer structures. A higher content typically indicates a higher degree of structural aggregation, with the network structure being primarily three-dimensional or ring-shaped. This may be because as the content of Fe_x_O_y_ increases, the contents of the network-modifying bodies, such as CaO and MgO, gradually decrease. Some of the FeO in Fe_x_O_y_ can act as network-modifying bodies to balance the charges in the system. The [AlO_4_] tetrahedral structure increases, and the network structure gradually transforms from tetrahedral monomers and island-like structures to three-dimensional and ring-shaped structures, resulting in an increased degree of polymerisation. 

In summary, when the Al_2_O_3_ content is 10–12% and the Fe_x_O_y_ content is 20%, the composition of glass evolves from simple to complex, promoting the improvement of the polymerisation degree and enhancing the thermal stability of the structure. This finding is consistent with the fitting results of the MD software, proving that controlling the Fe_x_O_y_ content can have a positive impact on the thermal stability of the IOW fibres. 

### 3.6. SEM Analysis of Rock Wool Fibers

**Figure 10:**
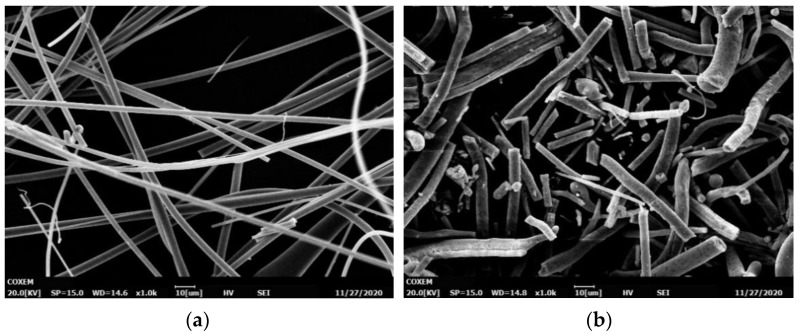
Microscopic morphology of rock wool fibres. (**a**) Iron ore waste rock wool fibres; (**b**) ordinary rock wool fibres.

Figure 10 represents the microscopic morphology of (a) iron ore waste rock wool and (b) ordinary rock wool observed by SEM after 800 °C high-temperature treatment.

It can be seen that the iron ore waste rock wool fibres’ surfaces are relatively smooth, a small amount of white material appears, and the changes in the fibres’ diameters are not large. Under the electron microscope at 1000×, it was observed that the damaged fibres were distributed in short rods and did not have a long fibrous structure. The elongated fibres became powdery, and the surface was not smooth with the generation of pore defects, while the fibres’ volume had a tendency to expand. It was speculated that crystalline substances might have been produced inside the fibres, leading to the coarsening of the fibre diameter.

## 4. Conclusions

The high-temperature glass body of rock wool fibres is easily crystallised and crushed, and rock wool made from IOW rich in iron and aluminium is expected to improve its thermal stability.

In this study, the effects of Fe_x_O_y_ and Al_2_O_3_ on the refractory structure and Fe_x_O_y_-Al_2_O_3_-SiO_2_-MgO-CaO composition of glass were investigated using MD software. The T_g_ values obtained through simulation and measurement were consistent, indicating that the MD simulation was reliable. The highest T_g_ values were 17–19% for Fe_x_O_y_ content and 10–13% for Al_2_O_3_ content; thus, IOW has a higher T_g_ and better thermal stability than ordinary rocks. With an increase in the Fe_x_O_y_ content, the bond lengths of Si-O and Al-O increased, the average CN remained at approximately 4, and the amount of bridge oxygen increased. Simultaneously, the network structure gradually transformed from tetrahedral monomers and island-like structures to three-dimensional and ring-like structures, making it more polymerised. These results prove that the Fe_x_O_y_-Al_2_O_3_-SiO_2_-MgO-CaO structure is more stable when the Al_2_O_3_ content is 10–12% and the Fe_x_O_y_ content is close to 20%, resulting in a higher T_g_.

In summary, an increase in the Fe_x_O_y_ content can improve the thermal stability of IOW fibres based on an Fe_x_O_y_-Al_2_O_3_-SiO_2_-MgO-CaO glass structure. In particular, when the Al_2_O_3_ content is 10–13% and the Fe_x_O_y_ content is 17–19%, the thermal stability of the IOW rock glass reaches its optimal level. The results of this study not only provide data in support of the preparation of high-thermal-stability fibres from IOW, but also offer a new approach to the improvement of the refractory properties of rock wool fibres based on the Fe_x_O_y_-Al_2_O_3_-SiO_2_-MgO-CaO system. 

## Figures and Tables

**Table 1:** The XPS Gaussian peak resolution of IOW rocks.

**IOW rock**	**Fe 2p_3/2_(eV)**		**Satellite peaks (eV)**		**Fe 2p_1/2_(eV)**		**Fe^2+^/Fe^3+^**
	**Fe^2+^**	**Fe^3+^**	**Fe^2+^**	**Fe^3+^**	**Fe^2+^**	**Fe^3+^**	
	**709.75**	**711.08**	**714.35**	**715.94**	**722.89**	**724.55**	**1/3**

**Table 2:** Composition and atomic numbers of IOW fibres based on FAMSC structure.

Sample	Mole Fraction (%)		Atomic Number							Total
	Fe_x_O_y_	Al_2_O_3_	Fe^2+^	Fe^3+^	Mg^2+^	Si^4+^	Al^3+^	Ca^2+^	O^2−^	
MX1	20%	10%	120	360	240	950	496	180	3655	6000
MX2	10%	20%	60	180	480	950	496	180	3655	6000
MX3	10%	10%	60	180	240	1085	566	206	3663	6000
MX4	15%	15%	90	270	360	950	496	180	3655	6000
MX5	15%	10%	90	270	240	1017	531	193	3659	6000
MX6	10%	15%	60	180	360	1017	531	193	3659	6000
MX7	13.33%	13.33%	80	240	320	995	519	188	3657	6000
MX8	16.67%	11.67%	100	300	280	972	508	184	3656	6000
MX9	11.67%	16.67%	70	210	400	972	508	184	3656	6000
MX10	11.67%	11.67%	70	210	280	1040	543	197	3660	6000

**Table 3:** Average bond length (Ri) of different atom pairs (Å).

Bond	Si-O	Al-O	Fe-O(Fe_2_O_3_)	Ca-O	Mg-O	Fe-O(FeO)
Bond length	1.66	1.78	1.89	2.55	2.43	2.1

## Data Availability

The raw data supporting the conclusions of this article will be made available by the authors on request.
